# Deep sequencing uncovers commonality in small RNA profiles between transgene-induced and naturally occurring RNA silencing of chalcone synthase-A gene in petunia

**DOI:** 10.1186/1471-2164-14-63

**Published:** 2013-01-30

**Authors:** Megumi Kasai, Hideo Matsumura, Kentaro Yoshida, Ryohei Terauchi, Akito Taneda, Akira Kanazawa

**Affiliations:** 1 Research Faculty of Agriculture, Hokkaido University, Sapporo, 060-8589, Japan; 2 Gene Research Center, Shinshu University, Ueda, 386-8567, Japan; 3 Iwate Biotechnology Research Center, Kitakami, 024-0003, Japan; 4 Graduate School of Science and Technology, Hirosaki University, Hirosaki, Aomori, 036-8561, Japan

**Keywords:** Chalcone synthase, Cosuppression, Deep-sequencing analysis, Flower color pattern, Naturally occurring RNA silencing, Short interfering RNA

## Abstract

**Background:**

Introduction of a transgene that transcribes RNA homologous to an endogenous gene in the plant genome can induce silencing of both genes, a phenomenon termed cosuppression. Cosuppression was first discovered in transgenic petunia plants transformed with the *CHS-A* gene encoding chalcone synthase, in which nonpigmented sectors in flowers or completely white flowers are produced. Some of the flower-color patterns observed in transgenic petunias having *CHS-A* cosuppression resemble those in existing nontransgenic varieties. Although the mechanism by which white sectors are generated in nontransgenic petunia is known to be due to RNA silencing of the *CHS-A* gene as in cosuppression, whether the same trigger(s) and/or pattern of RNA degradation are involved in these phenomena has not been known. Here, we addressed this question using deep-sequencing and bioinformatic analyses of small RNAs.

**Results:**

We analyzed short interfering RNAs (siRNAs) produced in nonpigmented sectors of petal tissues in transgenic petunia plants that have *CHS-A* cosuppression and a nontransgenic petunia variety Red Star, that has naturally occurring *CHS-A* RNA silencing. In both silencing systems, 21-nt and 22-nt siRNAs were the most and the second-most abundant size classes, respectively. *CHS-A* siRNA production was confined to exon 2, indicating that RNA degradation through the RNA silencing pathway occurred in this exon. Common siRNAs were detected in cosuppression and naturally occurring RNA silencing, and their ranks based on the number of siRNAs in these plants were correlated with each other. Noticeably, highly abundant siRNAs were common in these systems. Phased siRNAs were detected in multiple phases at multiple sites, and some of the ends of the regions that produced phased siRNAs were conserved.

**Conclusions:**

The features of siRNA production found to be common to cosuppression and naturally occurring silencing of the *CHS-A* gene indicate mechanistic similarities between these silencing systems especially in the biosynthetic processes of siRNAs including cleavage of *CHS-A* transcripts and subsequent production of secondary siRNAs in exon 2. The data also suggest that these events occurred at multiple sites, which can be a feature of these silencing phenomena.

## Background

RNA silencing refers collectively to diverse RNA-mediated pathways of nucleotide-sequence-specific inhibition of gene expression. RNA silencing of genes is induced by the presence of double-stranded RNA (dsRNA) homologous to the genes. The dsRNAs are processed into small RNAs, especially 21- to 24-nulceotide (nt) short interfering RNAs (siRNAs), by a dsRNA-specific ribonuclease, Dicer or Dicer-like (DCL) proteins [1,2]. In *Arabidopsis*, DCL2, DCL3 and DCL4 produce 22-, 24- and 21-nt siRNAs, respectively [3]. The siRNAs are incorporated into Argonaute (AGO) proteins and serve as a guide for sequence-specific cleavage of a target RNA, leading to posttranscriptional gene silencing (PTGS) [4,5]. Transcriptional repression can also be induced by dsRNA, which contains a sequence homologous to a gene promoter and can trigger cytosine methylation on the promoter in the nuclear DNA resulting in transcriptional gene silencing (TGS) [6-8]. Like siRNAs, small RNAs called microRNAs (miRNAs) also negatively regulate the expression of endogenous genes through either RNA cleavage or the arrest of translation, which is another pathway of RNA silencing [1,9]. Small RNA (miRNA or siRNA)-mediated cleavage of an RNA can trigger the production of 21-nt secondary siRNAs either upstream or downstream of the original target site, a phenomenon called transitivity [4]. In *Arabidopsis*, small RNA-mediated cleavage can trigger conversion of the targeted RNA to dsRNA by RNA-dependent RNA polymerase 6 (RDR6), which is then cleaved into 21-nt phased siRNAs by DCL4. These siRNAs can include those termed trans-acting siRNAs (tasiRNAs), which silence other gene(s) *in trans*[10-12]. Small RNAs of 22 nt trigger RDR6-dependent secondary siRNA production [13,14]. A recent study indicated that the presence of 22-nt RNA in either strand of the small RNA duplex is sufficient for this reaction [15].

Overexpression of the *chalcone synthase-A* (*CHS-A*) gene under the control of the cauliflower mosaic virus (CaMV) 35S promoter and the nopaline synthase (NOS) terminator causes the production of white sectors or completely white flowers in transformed petunia (*Petunia hybrida*) plants [16,17]. This system was the first example of RNA silencing induced by a transgene. In these transgenic petunia plants, silencing of both the *CHS-A* transgene and endogenous *CHS-A* gene was induced, so that the event was termed cosuppression [16]. The production of the wild-type pigment is inhibited because chalcone synthase performs an essential step in the biosynthesis of anthocyanins. Various silencing patterns in the petal tissues have been observed in the petunia *CHS-A* silencing system [18,19]. Because it induces visibly altered phenotypes, *CHS-A* silencing in petunia is a model system to study RNA silencing [20]. Based on the inhibition of pigmentation in flower petals, Sijen et al. demonstrated that a transgene that expresses dsRNA corresponding to the transcribed region and the promoter region induced PTGS and TGS, respectively [8]. In our recent study, we used a virus vector and succeeded in inducing heritable TGS of the endogenous *CHS-A* gene, thereby produced a plant that does not carry a transgene but has altered traits [21,22].

Some of the flower-color patterns observed in transgenic petunias having cosuppression of the *CHS-A* genes resemble those in nontransgenic varieties [18]. One such variety is Red Star, which produces bicolor flowers having a star-type red and white pattern. As expected from the phenotypic similarity with the flowers of *CHS-A* cosuppressed plants, the flower color pattern in Red Star was in fact demonstrated to be due to sequence-specific degradation of the *CHS-A* RNA in the white sectors [23]. Petunia breeding was launched in the 1830s by crossing among wild species [18]. The generation of the star-type petunia flowers as a consequence of hybridization between plant lines suggests that the RNA silencing ability can be conferred via the shuffling of genomes that differ slightly from each other [20]. Similar naturally occurring RNA silencing has been reported for a picotee-type variety of petunia, which has nonpigmented sectors in the outer edge of the petal tissues [24], and for other plants such as rice [25], soybean [26-29], maize [30] and dahlia [31].

Cosuppression has been thought to be caused by a couple of mechanisms. It can be induced when multiple transgenes are integrated into the same site in the genome in an inverted orientation and fortuitous read-through transcription over the transgenes produces dsRNA homologous to an endogenous gene in the genome, a pathway termed inverted repeat (IR)-PTGS. When sense transcripts from a transgene trigger cosuppression through RNA degradation, the pathway is referred to as sense (S)-PTGS [4]. A model for S-PTGS proposes that transgene-derived aberrant RNAs that lack a poly(A) tail or 5^′^ capping are used as a template for RDR6 to produce dsRNA, thereby triggering PTGS [4]. An alternative scenario is that nuclear-accumulated sense transcripts form imperfect hairpin structures, which resemble miRNA precursors, are processed into small RNAs and function as a trigger for RNA degradation via RDR-catalyzed synthesis of dsRNA, resulting in PTGS [32].

Our previous data indicated that *CHS-A* cosuppression is induced by a high level of transcription of the *CHS-A* transgene, shown by the fact that *CHS-A* cosuppression is induced when the *CHS-A* transgene is transcribed by the CaMV 35S promoter but not when the transcription from the promoter is repressed by epigenetic changes involving spontaneous cytosine methylation of the promoter [33]. These observations are consistent with the threshold model for induction of RNA degradation, which was first suggested on the basis of a viral RNA analysis: viral RNA degradation is triggered when the amount of viral RNA exceeds a certain level in plant cells [34]. This notion is also consistent with the fact that the frequency of cosuppression in petunia is correlated with the strength of the promoter of the *CHS-A* transgene [35]. Thus, *CHS-A* cosuppression can be triggered when a particular RNA, e.g., *CHS-A* primary transcripts or some other RNA molecule(s) derived from them, exceed a certain level. However, neither the RNA molecule(s) nor the sensing mechanism(s) of the threshold is known.

A potential trigger for *CHS-A* cosuppression in petunia has been suggested on the basis of a deep sequencing analysis of *CHS-A* siRNAs [36]. Two abundant siRNAs in antisense polarity, termed phy-siR1 and phy-siR2, were detected in a cosuppressed line. On the basis of the presence of these siRNAs with phased siRNAs, the authors proposed that these two siRNAs guide *CHS-A* mRNA cleavage and initiate the generation of phased siRNAs, leading to cosuppression. On the other hand, *CHS-A* siRNA profiles in another cosuppressed transgenic line having inverted repeat T-DNA [37] or a petunia variety that produces picotee-type flowers [24] indicated the presence of multiple abundant siRNAs. At present, whether the population of siRNAs detected in one *CHS-A* cosuppressed line is common to different *CHS-A* cosuppressed lines or *CHS-A* naturally silenced lines is not known. Moreover, no insight into a general mechanism(s) of cosuppression in terms of siRNA production has been presented in any plant species.

To address these questions, here we analyzed *CHS-A* siRNA populations from silenced and nonsilenced tissues of a transgenic line having *CHS-A* cosuppression and a non-transgenic variety Red Star in detail. We show that multiple abundant siRNAs from *CHS-A* exon 2 are produced in the silenced tissues in both silenced lines. We also found profound commonality in siRNA production in the silenced tissues of the cosuppressed line and Red Star, which suggests the presence of a common mechanism of RNA degradation that likely depends on an evolutionary conserved feature in exon 2 of the *CHS-A* gene.

## Results

**Figure 1:**
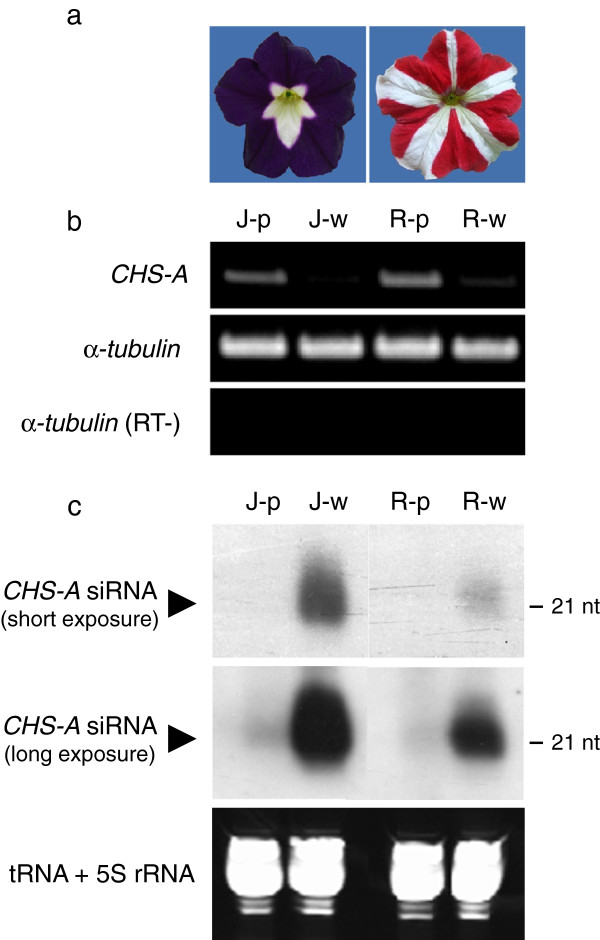
**Detection of *CHS-A *mRNA and siRNAs in J-type and Red Star flowers.** (**a**) Flower phenotypes of J-type (left) and Red Star (right) plants. (**b**) Steady state mRNA levels of the *CHS-A* gene in the white and pigmented petal tissues examined by RT-PCR. J-p, pigmented portions of petals in J-type; J-w, white portions of petals in J-type; R-p, pigmented portions of petals in Red Star; R-w, white portions of petals in Red Star. Transcripts of *α-tubulin* gene were amplified as a positive control. A reaction mixture without reverse transcriptase was used as a control to confirm that no amplification occurred from genomic DNA contamination of the RNA sample (RT-). (**c**) Detection of *CHS-A* siRNAs by Northern blot analysis. Same tissues were used as in the RT-PCR. Hybridization signals obtained with two exposure durations are shown. Ethidium-bromide-stained tRNA and 5S rRNA bands are shown below the panels to show that an equal amount of the small RNA fraction was loaded.

We analyzed the mRNA and siRNAs of the *CHS-A* gene in the white and pigmented portions of petal tissues of petunia plants that have cosuppression or naturally occurring RNA silencing of the *CHS-A* gene. The *CHS-A* cosuppressed line contains a single copy of the *CHS-A* transgene and produces petals with a white and purple pattern. The size of the white portions is variable, but they are invariably centered on the junctions between petals; hence, the pattern is called the junction pattern [19] (Figure 1a, left). The bicolor petals of nontransgenic variety Red Star have a star-type white and red pattern: the white sector forms along the veins in the center of each petal (Figure 1a, right). In the white petal tissues of both the junction-type (J-type) and Red Star plants, *CHS-A* mRNA was barely detected (Figure 1b) but *CHS-A* siRNAs accumulated (Figure 1c), confirming the occurrence of *CHS-A* RNA degradation [23].

### Mapping of siRNAs on the *CHS-A* gene in a *CHS-A* cosuppressed line

**Figure 2:**
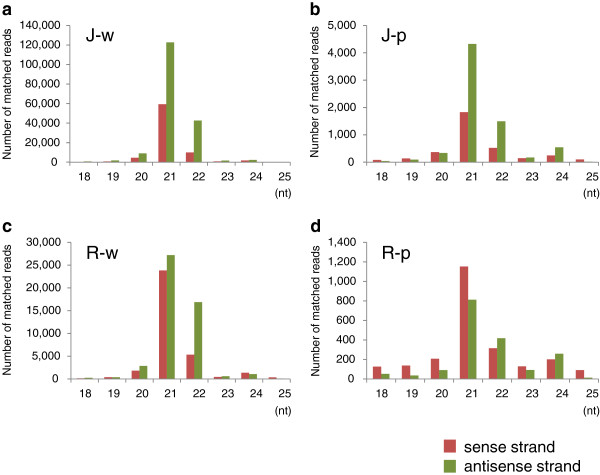
**Frequency of siRNAs between 18 and 25 nt mapped in the *CHS-A *gene from floral tissues of petunia.** (**a**) White and (**b**) pigmented tissues of J-type plants, (**c**) white and (**d**) pigmented tissues of Red Star plants. Number of siRNAs mapped on the sense strand (red bars) and antisense strand (green bars) are indicated.

**Figure 3:**
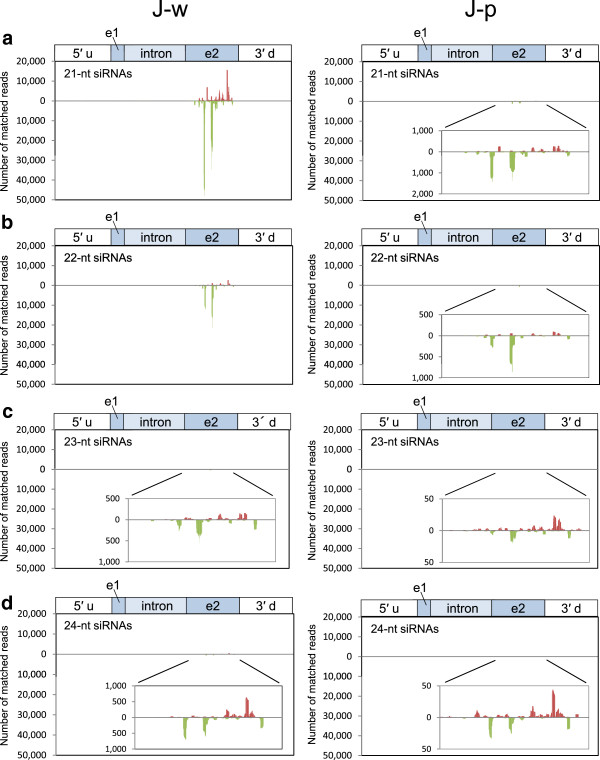
**Position and abundance of siRNAs mapped on the *CHS-A *gene region in J-type plants.** Data for (**a**) 21-nt, (**b**) 22-nt, (**c**) 23-nt and (**d**) 24-nt siRNAs in the white (J-w) and pigmented (J-p) petal tissues are shown. Bars above and below the *x*-axis indicate siRNAs mapped on the sense and antisense strands, respectively. A detail of the siRNA data in exon 2 is inserted when the level was very low. 5′ u, 5′ upstream region; e1, exon 1; e2, exon 2; 3′ d, 3′ downstream region.

We analyzed siRNAs in J-type plants by deep sequencing technology. Of 21,138,355 reads, 261,197 reads matched the *CHS-A* gene region in the white portions of petals in J-type plants (Table 1). The size distribution of siRNAs mapped in the *CHS-A* gene region revealed the predominance of siRNAs of 21 nt and 22 nt, especially 21 nt, for both sense and antisense strands in this plant line (Figure 2a). This result indicates that *CHS-A* siRNAs are predominantly produced by the function of DCL4 orthologue(s). The position and abundance of the 21-nt to 24-nt siRNAs mapped in the *CHS-A* gene region are shown in Figure 3. Almost all the siRNAs were mapped to exon 2 of the *CHS-A* gene region (see, for example, Figure 3a, b). There is uneven distribution of siRNA within exon 2, indicating the presence of multiple hot spots for siRNA production. *CHS-A* siRNAs were also detected in the purple portions of petals in J-type plants, although the level of siRNAs was 1/30 of the level in the white tissues (Figure 2a, b). The presence of siRNAs at a low level in the pigmented petal tissues was also shown by the Northern blot analysis (Figure 1c, see “long exposure”).

**Table 1:** **Number of siRNA reads mapped in the *CHS-A *gene region**

**Read statistic**	**J-w**	**J-p**	**R-w**	**R-p**
Total reads analyzed	21,138,355	26,817,315	21,318,490	25,612,671
Total reads mapped in *CHS-A* region				
Sense strand	78,684	3,672	33,958	2,716
Antisense strand	182,513	7,058	49,627	1,812
Total	261,197	10,730	83,585	4,528

Abbreviations: J-p, pigmented portions of J-type petals; J-w, white portions of J-type petals; R-p, pigmented portions of Red Star petals; R-w, white portions of Red Star petals.

**Figure 4:**
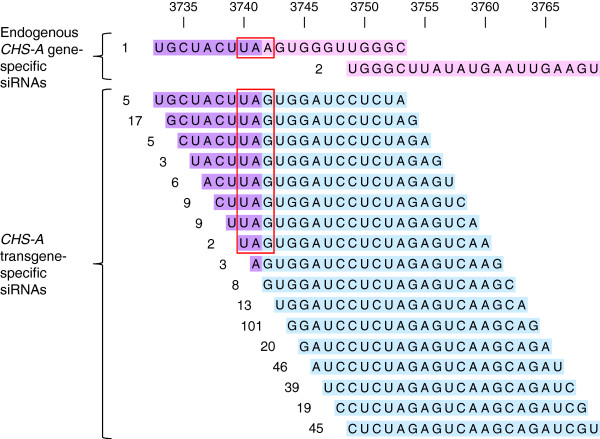
**siRNAs derived from the endogenous *CHS-A *gene and the *CHS-A *transgene.** Sense strand 21-nt siRNAs that have nucleotide sequences specific to either the endogenous *CHS-A* gene (*CHS-A* endogene) or the *CHS-A* transgene in the vicinity of translational termination codon (indicated by red boxes) are shown. Nucleotide sequences are described in the 5′ to 3′ direction from left to right. Read numbers of siRNAs are shown to the left. Nucleotide positions in the *CHS-A* endogene are shown at the top. siRNA sequences common to the *CHS-A* endogene and *CHS-A* transgene are colored in purple, and those specific to the *CHS-A* endogene and *CHS-A* transgene are colored in pink and blue, respectively. Similar patterns were also observed for antisense strand in J-type. No siRNA was detected in this region in wild-type V26 plants (data not shown).

The endogenous *CHS-A* gene and *CHS-A* transgene have different nucleotide sequences in the 3′ untranslated region. siRNAs specific to the endogenous *CHS-A* gene and those specific to the *CHS-A* transgene were both detected (Figure 4), which indicates that mRNAs derived from the endogenous *CHS-A* gene and *CHS-A* transgene are both degraded via RNA silencing pathways. The number of siRNA mapped in this region was higher for the *CHS-A* transgene than for the endogenous *CHS-A* gene (Figure 4).

### Mapping of siRNAs on the *CHS-A* gene in a non-transgenic variety

**Figure 5:**
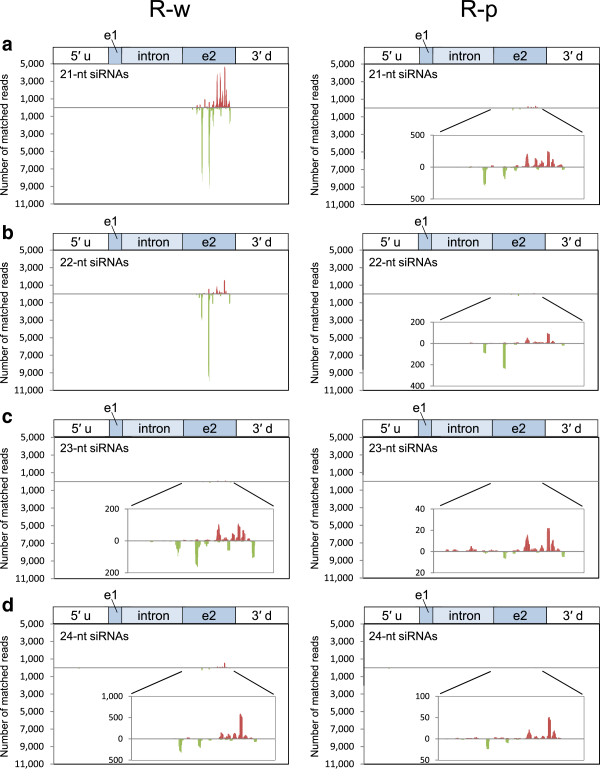
**Position and abundance of siRNA mapped on the *CHS-A *gene region in Red Star plants.** For more details, see Figure 3 legend.

The production of *CHS-A* siRNAs was also analyzed in petal tissues of Red Star. In the white portions of petals, 21-nt and 22-nt siRNAs were predominant (Figure 2c). The production of siRNAs was confined to exon 2, which included multiple highly abundant siRNAs (Figure 5), as observed for the white portions of J-type plants. Similarly, *CHS-A* siRNAs were also detected in red portions of Red Star flowers at a very low level (1/20–1/40 of the level in white tissues; Figure 2c, d). The read number indicated that more *CHS-A* siRNAs were detected in the white petal tissues of J-type plants than in those of Red Star plants (Table 1; Figure 2a, c). These results are consistent with the difference in the signal intensity in the Northern blot analysis (Figure 1c).

### The presence of siRNAs mapped in the vicinity of the intron–exon 2 boundary

**Figure 6:**
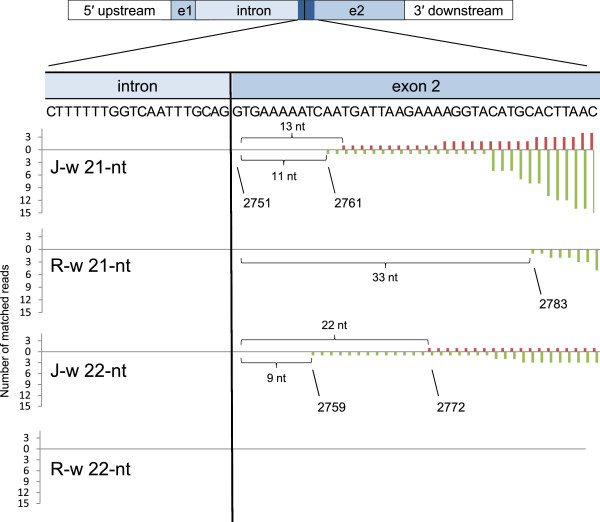
**Position and abundance of siRNAs mapped in the boundary region between intron and exon2 of the endogenous *CHS-A *gene.** Data for 21-nt and 22-nt siRNAs in the white petal tissues of J-type (J-w) and Red Star (R-w) plants are shown. DNA sequence in the intron–exon 2 boundary (20 nt of intron and 40 nt of exon 2; nucleotide positions 2731–2790) is shown. Bars above and below the *x*-axis indicate siRNAs mapped on the sense and antisense strands, respectively. Nucleotide positions of the 5′ end of exon 2, and those of the 3′ ends of antisense siRNAs mapped in this region are indicated. No 22-nt siRNA was mapped in this region in R-w.

Because the distribution of *CHS-A* siRNAs was confined to exon 2 in both J-type and Red Star plants, we had a close look at the mapping of siRNAs in the boundary between intron and exon 2. The 21-nt siRNAs mapped closest to intron in exon 2 were 13 nt and 11 nt distant from the intron–exon 2 boundary in the white portions of J-type plants for sense and antisense strands, respectively (Figure 6). Similarly, the siRNA mapped closest to the boundary was 51 nt (data not shown) and 33 nt (Figure 6) distant from the boundary in the white portions of Red Star for sense and antisense strands, respectively. In addition, 22-nt siRNAs of both sense and antisense strands were mapped at similar positions (22 nt and 9 nt distant from the boundary, respectively) in the J-type (Figure 6). Thus, the 5′ end of siRNA production in exon 2 was very close to the intron–exon 2 boundary in both J-type and Red Star plants.

### Commonality in the abundance of siRNAs between J-type and Red Star plants

**Figure 7:**
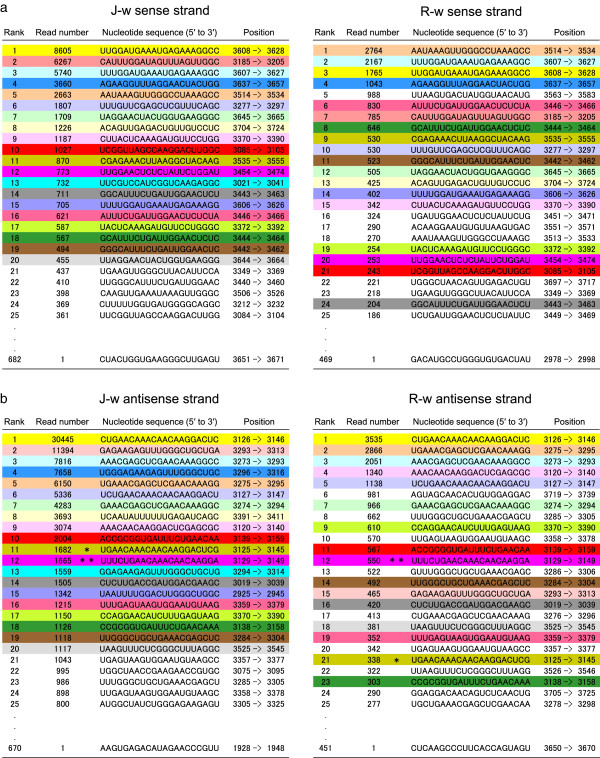
**Highly abundant 21-nt siRNAs in the white portions of petals in J-type and Red Star plants.** The order of siRNAs of sense (**a**) and antisense (**b**) strands was arranged according to the number of reads in deep-sequencing analysis. J-w and R-w refer to the siRNAs detected in the white portions of J-type and Red Star plants, respectively. Twenty most abundant siRNAs in J-w are colored, and the siRNAs of the same sequence in Red Star are colored the same. Note that most of the highly abundant siRNAs in J-type plants are also highly abundant in Red Star plants. Phy-siR1 and phy-siR2 [36] are indicated by single and double asterisks, respectively.

We compared the read number of 21-nt siRNAs between J-type and Red Star plants. We found that siRNAs highly abundant in the white portions of J-type plants were also highly abundant in the white portions in Red Star, and vice versa. For example, 18 of the 20 most abundant siRNAs of the sense strand (in 682 siRNA species) detected in the white portions of J-type plants were found within the 24 most abundant siRNAs (in 469 siRNA species) detected in the white portions of Red Star flowers (Figure 7a). Similarly, 16 of the 20 most abundant siRNAs of the antisense strand (in 670 siRNA species) detected in the white portions of J-type plants were found within the 23 most abundant siRNAs (in 451 siRNA species) in the white portions of Red Star plants (Figure 7b). Most strikingly, the same siRNA of antisense strand was most abundant in both J-type and Red Star plants (Figure 7b).

To compare the overall feature of siRNA production between J-type and Red Star plants, we analyzed the correlation in the rank of siRNAs based on the number of reads between J-type and Red Star plants. We calculated Spearman’s rank correlation coefficient using siRNAs that had more than five reads, which cover 97–99% of all siRNA reads (see “value B / value A” in Table 2); 214 sense and 180 antisense different siRNA species, which were detected in both J-type and Red Star plants, were used for the calculation. The results indicated that the siRNA ranks in J-type and Red Star plants are highly correlated with each other for both sense and antisense strands (for the sense strand, *r*_*s*_ = 0.723, *P* < 0.01; for the antisense strand, *r*_*s*_ = 0.852, *P* < 0.01) (Table 2). Taken together, these results indicate commonality between J-type and Red Star plants in terms of siRNA production.

**Table 2:** Reads and rank correlation of 21-nt siRNAs in white tissues of J-type and Red Star petals

**Read statistic**	** Sense strand**		** Antisense strand**	
	**J-w**	**R-w**	**J-w**	**R-w**
Total number of siRNA reads (value A)	59,386	23,840	122,753	27,197
Total number of reads for siRNA species with >5 reads (value B)	58,705	23,287	122,033	26,646
Value B / value A	0.989	0.977	0.994	0.980
Total number of siRNA species	682	469	670	451
Number of siRNA species with >5 reads	374	219	337	182
Number of siRNA species with >5 reads in both J-w and R-w	214		180	
Rank correlation coefficient (*r*_*s*_ )	0.723^a^		0.852^a^	

^a^*P* < 0.01.

Abbreviations: J-w, white portions of J-type petals; R-w, white portions of Red Star petals.

A similar correlation in the rank of siRNAs between J-type and Red Star plants was also detected for 22-nt siRNAs (Additional file 1: Figure S1). For example, the two most abundant siRNAs were common to J-type and Red Star plants for both sense and antisense strands.

### Commonality in the production of phased siRNAs

**Figure 8:**
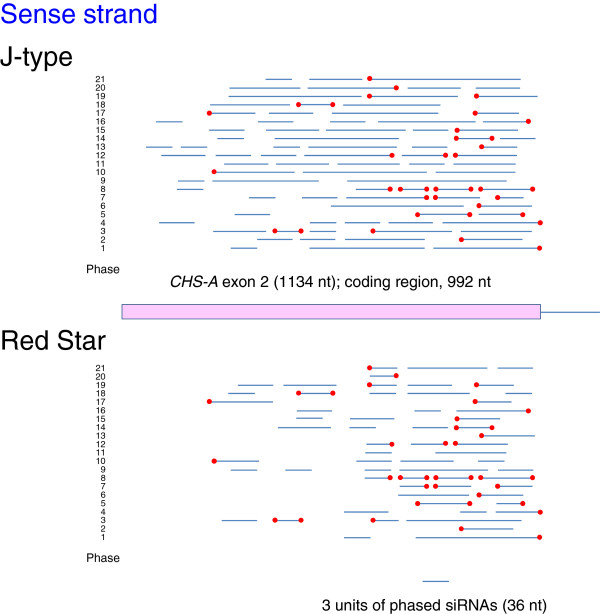
**Phased siRNAs from sense strand of *CHS-A *exon 2.** Data in upper and lower panels were obtained from white portions of J-type and Red Star petals, respectively. Presence/absence of 21-nt siRNAs was analyzed in 21 phases independently. The results of each phase are marked 1–21: the first nucleotide of “phase 1” corresponds to the first and last nucleotides of the *CHS-A* reference sequence (see Methods) for sense and antisense strands, respectively. Blue lines: regions producing phased siRNAs of three or more contiguous units. Red dots: 5′ or 3′ ends of phased-siRNA producing regions that are common to J-type and Red Star petals.

**Figure 9:**
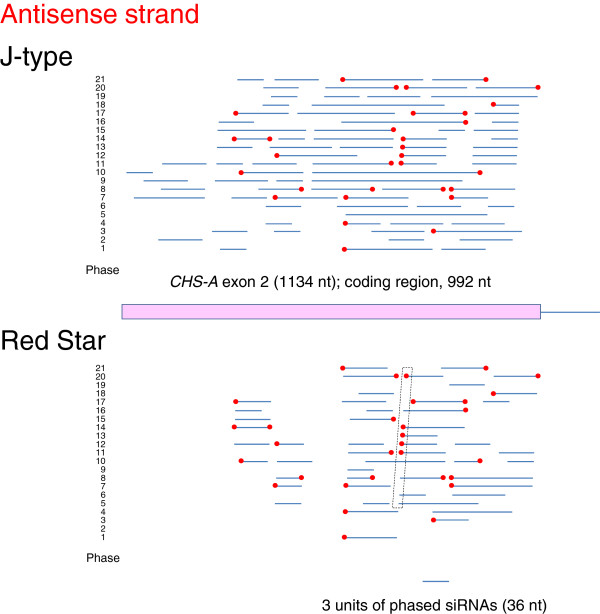
**Phased siRNAs from antisense strand of *CHS-A *exon 2.** The ends of phased siRNAs indicated by dotted lines are shown in detail in Figure 12. For other details, see legend to Figure 8.

In *Arabidopsis*, cleavage of transcripts by a small RNA can result in in-phase generation of 21-nt secondary siRNAs by DCL4 after production of dsRNA by RDR6 [11,12]. To detect phased siRNAs in the J-type and Red Star plants, we mapped siRNAs of the *CHS-A* gene independently in 21 different phases. Figures 8 and 9 show the distribution of 21-nt phased siRNAs that are contiguous for three or more units in each phase in exon 2. These phased siRNAs were detected in all 21 phases in both J-type and Red Star plants for both sense (Figure 8) and antisense (Figure 9) strands except for “phase 2” of the antisense strand in Red Star (Figure 9).

**Figure 10:**
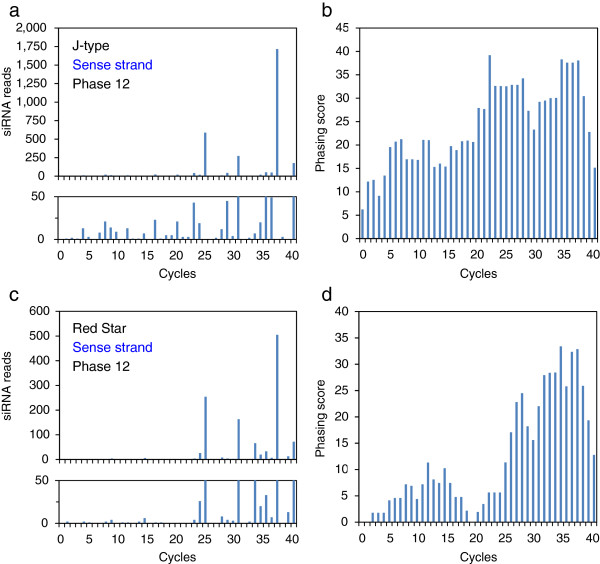
**Abundance of 21-nt siRNAs and phasing scores of sense strand.** Data of phase 12 of sense strand siRNAs, in which total number of contiguous siRNA units was highest in J-type (see Figure 8), are shown. **a**, siRNA reads in J-type; **b**, phasing score of the data in panel a; **c**, siRNA reads in Red Star; **d**, phasing score of the data in panel c. In panels a and c, a close up of the graphs up to 50 reads are shown below. Cycle 1 corresponds to the phased siRNA mapped at the upstream end of contiguous units in J-type (Figure 8). Phasing scores are calculated according to Howell et al. (2007).

**Figure 11:**
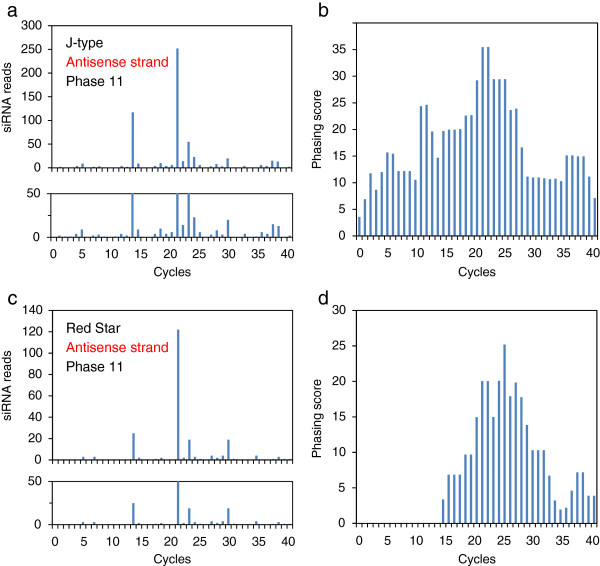
**Abundance of 21-nt siRNAs and phasing scores of antisense strand.** Data of phase 11 of antisense strand siRNAs, in which total number of contiguous siRNA units was highest in J-type (see Figure 9), are shown. Cycle 1 corresponds to the phased siRNA mapped at the upstream end of contiguous units in J-type (Figure 9). For other details, see legend to Figure 10.

Phased siRNAs were distributed more widely in J-type plants than in Red Star plants: the 5′ end of phased siRNA-producing region in J-type was 151-nt and 254-nt upstream of that in Red Star for sense and antisense strands, respectively, while the 3′ end of phased siRNA-producing region encompassed the 3′ end of the *CHS-A* coding region in both J-type and Red Star plants. Both siRNA reads and phasing scores were consistent with a wider distribution of phased siRNAs in J-type than in Red Star (Figures 10 and 11). The maximum number of contiguous units was 19, which covers a 399-nt region (in J-type antisense strand, phase 10) (Figure 9). Some of the 5′ ends or 3′ ends of the regions that produced phased siRNAs in Red Star plants were conserved in J-type plants; of the 62 regions that produced phased siRNAs for sense strand in Red Star plants (Figure 8, indicated by blue lines), 21 of the 5′ ends and 16 of the 3′ ends were conserved in J-type plants (Figure 8, indicated by red dots). Similarly, of the 50 regions that produced phased siRNAs for antisense strand in Red Star plants, 19 of the 5′ ends and 12 of the 3′ ends were conserved in J-type plants (Figure 9). We also found that 21-nt siRNAs mapped in the vicinity of intron–exon 2 boundary in the antisense strand in J-type plants (Figure 6) were phased siRNAs (Figure 9; phase 10).

Overall, these data indicate that phased siRNAs were produced in multiple phases at multiple sites over exon 2 in both J-type and Red Star plants. The presence of common ends of phased siRNAs suggests that the mechanism(s) of the production of phased siRNAs, including the sites of RNA cleavage to initiate phased siRNA production, is considerably conserved between these plants for both sense and antisense strands.

## Discussion

### Small RNA profiles suggest a common mechanism of RNA degradation in cosuppression and naturally occurring RNA silencing of the *CHS-A* gene

We found that various features of small RNA production in white petal tissues are common to J-type and Red Star plants: predominant size class, exon-2-specific production, the highly abundant species, and in-phase production of siRNAs. Multiple abundant 21-nt or 22-nt siRNAs can be produced from DCL cleavage of secondary-structured nascent *CHS-A* transcripts. They may cleave *CHS-A* RNA with AGO orthologue(s) to trigger secondary siRNA production. Alternatively, these abundant siRNAs can be a product of DCL cleavage of dsRNAs synthesized by an RDR6 orthologue(s) from the nascent transcripts or AGO-cleaved transcripts (Additional file 2: Figure S2). It is also possible that the dsRNAs are formed by intermolecular RNA interaction [38]. In these scenarios, differences in the abundance of siRNAs reflect differences in the efficiency of these biosynthetic processes or in the stability of siRNAs possibly mediated by association with AGO orthologue(s). The presence of common siRNAs suggests that sequence and/or structural preference in these processes is highly conserved in the two silencing systems.

**Figure 12:**
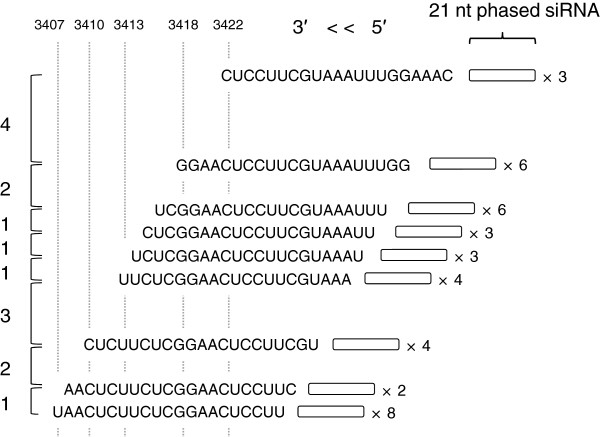
**Observed features of the ends of phased siRNAs.** An example of phased 21-nt siRNAs whose ends were mapped at positions that were very close to each other in Red Star petals. Nucleotide sequences of siRNAs located at the 5′ end of phased siRNAs are shown. Open boxes: observed phased siRNA units next to the sequences. Differences in the number of nucleotide between neighboring phases are indicated to the left.

Phased siRNAs of multiple phases were detected in this study. The presence of common ends of the regions that produced the phased siRNAs between J-type and Red Star plants suggests that the positions of the cleavages of *CHS-A* transcripts and subsequent production of secondary siRNAs are conserved. In addition, the mapping data suggested that phased siRNAs were produced from neighboring phases, the 5′ or 3′ end of which was mapped at positions that differed by one nucleotide (Figure 12). A mechanism that could allow this phenomenon is the production of siRNAs of more than one phase by a single cleavage, but no evidence for this scenario has been reported. Alternatively, the primary siRNAs that determine the initiation site of phasing might be produced from positions differed by one or a few nucleotides. The fact that highly abundant siRNAs were mapped at positions that are very close to each other (see below) is consistent with the notion that the primary siRNAs may be produced from such a limited place. Because of the presence of phased siRNAs of various phases at various regions of exon 2, we propose that, irrespective of the pathway of initial production of dsRNA, RNA cleavage at various sites that initiate production of secondary siRNAs can be a feature of both cosuppression and naturally occurring RNA silencing of the *CHS-A* gene (Additional file 2: Figure S2).

### Exon-2-specific production of siRNAs

In both J-type and Red Star plants, siRNA production was almost always confined to exon 2. Moreover, the 5′ end of siRNA production in exon 2 was very close to intron. These observations suggest that the primary event of *CHS-A* RNA degradation occurred in exon 2, and subsequent transitive RNA degradation did not reach the intron across the intron–exon 2 boundary.

It is possible that this phenomenon is associated with splicing. In fact, the presence of intron and/or splicing can suppress RNA silencing in plants [39,40]. In this regard, binding of factors involved in splicing, e.g., U2 auxiliary factors that bind to the 3′ splice site upon splicing [41] or splicing factors that remain associated with the exon–exon junction even after splicing is completed [42], might inhibit progression of dsRNA synthesis over the intron–exon boundary. However, in the white tissues of J-type plants not only the endogenous *CHS-A* gene transcripts but also the *CHS-A* transgene transcripts were degraded, while very few siRNAs were produced outside exon 2. These observations indicate that exon 2-specific production of siRNAs occurred even on transcripts lacking an intron. Therefore, there may be mechanism by which siRNA production from *CHS-A* transgene transcripts may be affected *in trans*, if splicing or spliceosome formation is involved in the exon-2-specific production of *CHS-A* siRNAs.

An alternative model to explain the exon-2-specific siRNA production is that the 5′ end of RNA degradation can be determined by an siRNA that targets a position in the vicinity of the intron–exon 2 boundary. The “two-hit trigger” model suggests that transitivity occurs in an RNA segment between two positions that are targeted by small RNAs [43]. According to this model, the observed siRNA production can be explained by the presence of siRNA that targets exon 2 in the vicinity of the intron–exon 2 boundary and another siRNA that targets a position downstream. Candidate siRNAs that may terminate degradation are those mapped in the vicinity of intron–exon 2 boundary (Figure 6).

Production of siRNAs that is essentially confined to exon 2 has also been observed for naturally occurring silencing of the *CHS* genes in soybean [28,44] and dahlia [31]. These results, together with the observations regarding the petunia *CHS-A* gene [24, 36, 37, this study], suggest that a conserved feature in exon 2 of the *CHS* gene across plant species, e.g., the secondary structure of transcripts and/or termination of transcription, is a key element involved in the induction of *CHS* RNA degradation. We mapped highly abundant siRNAs on the secondary structure of *CHS-A* RNA predicted by using m-fold software [45]. Some of the highly abundant siRNAs were mapped within limited regions that formed an incomplete dsRNA structure comprising both a stretch of base-pairing and an unpaired loop structure (Additional file 3: Figure S3). Such a structure is reminiscent of the fact that the presence of bulges adjacent to the cleavage site is important for processing primary miRNAs [46]. It is tempting to speculate that such a “partially opened” structure is preferred by DCL or RDR6 orthologue(s) and leads to the production of abundant siRNAs.

### Potential triggers of cosuppression and naturally occurring RNA silencing of the *CHS-A* gene

Among the cases of naturally occurring RNA silencing so far reported, a triggering mechanism has been suggested for only a few cases, all of which involve production of dsRNA either through read-through transcription of duplicated and rearranged genes [25,47,48] or through convergent transcription of an overlapping gene pair [49]. The presence of an inverted repeat comprising *CHS* genes or gene segments is correlated with *CHS* RNA silencing in soybean, and loss of such structures suppresses its induction in spontaneous mutants [28,29]. In petunia, the mechanism(s) responsible for naturally occurring *CHS-A* RNA silencing is not known, aside from the fact that the silencing occurs via RNA degradation that involves siRNA production [23]. A correlation between naturally occurring *CHS-A* RNA silencing that results in the star-type or picotee-type flower color pattern and the presence of two tandemly linked *CHS-A* genes has been reported in petunia [24]. However, these two *CHS-A* genes are separated by a long sequence (almost 7 kb), and a causative relationship between RNA silencing and the presence of the two copies of the *CHS-A* gene has not been presented.

For sense RNA-mediated silencing such as cosuppression in transgenic plants, a threshold sensing model, in which aberrant single-stranded RNA that accumulates beyond a critical level triggers its copying into dsRNA, has been suggested [50]. In fact, previous observations in *CHS-A* cosuppressed petunias are consistent with this notion [33,35]. Meanwhile, De Paoli et al. reported the presence of two extra-abundant 21-nt siRNAs of antisense polarity of *CHS-A*, phy-siR1 and phy-siR2, in a *CHS-A* cosuppressed petunia line and proposed that these siRNAs may trigger subsequent degradation of *CHS-A* transcripts [36]. On the other hand, we found that there are 21-nt siRNAs of both sense and antisense polarities that are more abundant than phy-siR1 and phy-siR2 (Figure 7; phy-siR1 and phy-siR2 are indicated by single and double asterisks, respectively). Moreover, no phased siRNAs whose end positions coincide with a cleavage in the middle of phy-siR1 or phy-siR2 were detected in this study (data not shown). These results, together with the presence of siRNAs in multiple phases, suggest that phy-siR1 and phy-siR2 are at least not the sole trigger for RNA degradation in different *CHS-A* cosuppressed lines, although circumstantial evidence indicates that RNA cleavages with these siRNAs can induce phased siRNA production [36]. The reason for the difference between our data and that of De Paoli et al. is not known at present, but we speculate that a slight difference in the developmental stage of the flowers could affect the composition of the siRNA population. Such a possibility needs to be examined, but can be excluded in the comparison between the J-type and Red Star plants of this study because flower tissues of an identical developmental stage were used for our analysis. Our data suggest that the *CHS-A* transcripts are cleaved at multiple, conserved positions in both J-type and Red Star plants. The siRNAs that guide these cleavages may include a potential trigger of RNA silencing. Whether a single cleavage of RNA can lead to extensive RNA degradation through RNA silencing pathways in these silencing systems is an issue to be addressed.

### The presence of siRNA at a low level in pigmented cells

We found that *CHS-A* siRNA was present in pigmented portions in both J-type and Red Star plants at a low level. On the other hand, an extremely low level (only 2 reads) of *CHS-A* siRNA was detected in 16,651,540 total reads for line V26 (data not shown), a wild-type plant that produces completely purple flowers and was used to produce J-type plants through the introduction of the *CHS-A* transgene. Therefore, the presence of *CHS-A* siRNAs in the pigmented petal tissues in J-type plants is associated with cosuppression that occurred in other cells of the petal tissue.

A likely explanation for the presence of *CHS-A* siRNA in pigmented cells is that RNA is degraded at a low rate in the pigmented cells. Alternatively, the siRNAs may migrate from cells that underwent PTGS through plasmodesmata. In either case, these results raise a novel possibility that a threshold level of *CHS-A* siRNAs might be associated with extensive RNA degradation in addition to the previous idea that an aberrant *CHS-A* primary transcript level constitutes such a threshold level. It would not be surprising that, taking into account the observed commonality in siRNA profiles between these two silencing systems, they share a common sensing mechanism for trigger RNAs.

## Conclusions

The present study revealed common features in siRNA production of the *CHS-A* gene between cosuppression in transgenic plants and naturally occurring silencing in nontransgenic plants of petunia. In both silencing systems, 21-nt and 22-nt siRNAs were the first- and the second-most abundant size classes, respectively. *CHS-A* siRNA production was confined to exon 2, indicating that *CHS-A* RNA is degraded through processes including cleavage and secondary siRNA production in this exon. Common siRNAs were detected in cosuppression and naturally occurring RNA silencing, whose ranks, according to the number of siRNAs in these plants, were correlated with each other. Highly abundant siRNAs were produced from multiple sites, many of which were common to the two silencing systems. Phased siRNAs were detected in multiple phases, and some of the ends of the regions that produced phased siRNAs were conserved. These results indicate mechanistic similarity between cosuppression and naturally occurring RNA silencing of the *CHS-A* gene, especially in the biosynthetic processes of siRNAs including cleavage of *CHS-A* transcripts and subsequent production of secondary siRNAs, which presumably depend on the nucleotide sequence and/or structural features of exon 2 RNA.

## Methods

### Plant materials

*Petunia hybrida* variety Red Star (Takii Seed Co., Japan) and a transgenic petunia line that produces junction-type flowers (J-type) [33] were used for analyses. The transgenic line is a descendent of the CHS223 line [19,51] and contains a single-copy *CHS-A* transgene. The white and the pigmented petal tissues of these plants were analyzed separately. Petal tissues were used at the developmental stage when the mRNA level of the *CHS-A* gene is highest [52].

### Isolation of total RNA and RT-PCR

Isolation of total RNA from flower tissues, cDNA synthesis, and RT-PCR were done as described previously [37]. The following primer pairs were used for the PCR: for the *CHS-A* gene, 4246 (5′-GGCGCGATCATTATAGGTTC-3′) and 5003 (5′-TTTGAGATCAGCCCAGGAAC-3′); for the *α-tubulin* gene, tub 125 F (5′-CAACTATCAGCCACCAACTG-3′) and tub 267R (5′-CACGCTTGGCATACATCAGA-3′).

### Northern blot analysis of siRNA

Low-molecular-weight RNA was isolated, and *CHS-A* siRNAs were detected by Northern blot analysis using a digoxigenin-labeled probe essentially as described by Goto et al. [53]. The following modifications were applied: RNA extraction buffer contained 100 mM Tris–HCl (pH 8.8), 20 mM EDTA, 200 mM NaCl and 4% *N*-lauroyl sarcosine; an RNA probe specific for *CHS-A* antisense RNA was labeled by *in vitro* transcription of the plasmid carrying a 0.44-kb region of the *CHS-A* gene [53] using DIG RNA labeling kit (Roche Applied Science, Basel, Switzerland) for use in hybridizations.

### Deep sequencing analysis of siRNA

Low-molecular-weight RNA was extracted from the petal tissues of flower buds before the buds opened (~4.5 cm long for J-type and ~5.0 cm long for Red Star). Tissues were frozen with liquid nitrogen and extracted with RNA extraction buffer containing 10 mM Tris–HCl (pH 7.5), 100 mM NaCl, 1 mM EDTA, and 1% (w/v) SDS. After extraction with phenol/chloroform, high-molecular-weight RNA was precipitated by mixing the aqueous phase with 1/3 volume of 8 M LiCl. After the solution was kept on ice overnight, the solution was centrifuged, and the nucleic acids in the supernatant were precipitated with ethanol. After centrifugation, the pellet was dissolved in water, and an equal amount of 20% polyethylene glycol (MW = 8000) was added to the solution to separate high-molecular-weight nucleic acids. The solution was held on ice for 1 h, then centrifuged, and low-molecular-weight RNA in the supernatant was precipitated with ethanol. After centrifugation, the pellet was dissolved in water and used for the following reactions. Low-molecular-weight RNA was ligated to 5′- and 3′-RNA adapters, reverse transcribed, and amplified by PCR using a Small RNA Sample Prep Kit (Illumina, San Diego, CA, USA) according to the manufacturer’s protocol except that we separated small RNAs by electrophoresis on a 3% agarose gel instead of an acrylamide gel. Nucleotide sequence of the amplified cDNA was analyzed using an Illumina Genome Analyzer. The adapter sequence was trimmed from the raw short-read data, and the resulting short reads (15–45 nt) were mapped to the nucleotide sequence of the *CHS-A* gene region (EMBL/GenBank/DDBJ database accession X14591), allowing only perfect matches. Nucleotide positions in this study correspond to those on this sequence. The secondary structure of *CHS-A* sense and antisense RNAs was predicted by m-fold software [45]. Correlation between the rank of the siRNA of J-type and Red Star plants was evaluated by Spearman’s rank correlation coefficient. Phased siRNAs were detected by independently mapping siRNAs of the *CHS-A* gene in 21 different phases. Calculation of phasing scores and assignment of scores to cycle position were done according to Howell et al. [54]. Nucleotide sequence data have been deposited in NCBI’s Gene Expression Omnibus and are accessible through GEO Series accession number GSE42965.

## Abbreviations

AGO: Argonaute; CaMV: Cauliflower mosaic virus; CHS: Chalcone synthase; DCL: Dicer-like; dsRNA: Double-stranded RNA; IR: Inverted repeat; J-type: Junction type; miRNA: microRNA; NOS: Nopaline synthase; PTGS: Posttranscriptional gene silencing; RDR: RNA-dependent RNA polymerase; S-PTGS: Sense-PTGS; siRNA: Short interfering RNA; tasiRNA: Trans-acting siRNA; TGS: Transcriptional gene silencing.

## Competing interests

The authors declare that they have no competing interests.

## Authors’ contributions

AK conceived and planned the study. MK did the experiments including RNA isolation, RT-PCR, Northern blot analysis, and preparation of small RNA cDNA library. HM, KY and RT carried out deep-sequencing of small RNAs. AT classified and mapped small RNA reads onto the gene sequence. MK and AK characterized small RNAs and drafted the manuscript. All authors read and approved the final manuscript.

## Supplementary Material

Additional file 1 Figure S1 Highly abundant 22-nt siRNAs in white portion of J-type and Red Star petals. The siRNAs of sense (a) and antisense (b) strands were ordered according to the number of reads in deep-sequencing analysis. J-w and R-w refer to the siRNAs detected in white portions of J-type and Red Star petals, respectively. The 10 most abundant siRNAs in J-w are colored, and the siRNAs of the same sequence in Red Star are colored the same. Note that most of the highly abundant siRNAs in J-type plants are also highly abundant in Red Star plants. Click here for file

Additional file 2 Figure S2 RNA cleavage at various sites that initiate production of siRNA can be a feature of siRNA production common to cosuppression and naturally occurring RNA silencing of the CHS-A gene. siRNAs are produced from DCL cleavage of secondary-structured nascent *CHS-A* transcripts or dsRNAs produced by RDR6 orthologue(s) from the nascent transcripts. These siRNAs then cleave the *CHS-A* RNA at the target site with AGO, which triggers RDR6-mediated dsRNA production and subsequent DCL cleavage that produces phased siRNAs. Click here for file

Additional file 3 Figure S3 Commonality of the siRNA hot spots between J-type and Red Star petals. Abundant siRNAs in the white tissues of J-type and Red Star petals are mapped on a secondary structure (a) and antisense (b) strands predicted by m-fold. Close ups of major hot spots are shown in windows, in which positions of nucleotides corresponding to siRNAs are marked by circles. Darker colors represent more total reads of siRNAs that contain the nucleotide. Mapped positions of siRNAs often overlapped, so that neighboring nucleotides had different colors. Nucleotide positions of abundant siRNAs mapped in each region are listed in the corresponding windows. Ranks of siRNA according to read number (see Figure 7) are in parentheses. Click here for file
