# Supplementary material for: Deep sequencing uncovers commonality in small RNA profiles between transgene-induced and naturally occurring RNA silencing of chalcone synthase-A gene in petunia
Source: BMC Genomics. 2013 Jan 30;14:63. doi: 10.1186/1471-2164-14-63 (PMC3608071; doi:10.1186/1471-2164-14-63)
Supplement: Additional file 1 Figure S1 — Highly abundant 22-nt siRNAs in white portion of J-type and Red Star petals. The siRNAs of sense (a) and antisense (b) strands were ordered according to the number of reads in deep-sequencing analysis. J-w and R-w refer to the siRNAs detected in white portions of J-type and Red Star petals, respectively. The 10 most abundant siRNAs in J-w are colored, and the siRNAs of the same sequence in Red Star are colored the same. Note that most of the highly abundant siRNAs in J-type plants are also highly abundant in Red Star plants. [file 1471-2164-14-63-S1.pdf]

Figure S1

a

J-w sense strand

Rank	Read number	Nucleotide sequence (5′ to 3′)	Position
1	2031	UUUUGGAUGAAAUGAGAAAGGC	3606 -> 3627
2	900	CUUUGUUCGAGCUCGUUUCAGC	3276 -> 3297
3	524	AAGAAGGUUUAGGAACUACUGG	3636 -> 3657
4	468	UUUGGAUGAAAUGAGAAAGGCC	3607 -> 3628
5	353	UUGGAACUCUCUAUUCUGGAUU	3454 -> 3475
6	232	UUCGGUUAGCCAAGGACUUGGC	3084 -> 3105
7	177	CAUUUGGAUAGUUUAGUUGGCC	3185 -> 3206
8	169	UUAGGAACUACUGGUGAAGGGC	3644 -> 3665
9	168	UGUUCGAGCUCGUUUCAGCAGC	3279 -> 3300
10	168	UUGGACCAAGUUGAAAUAAAGU	3500 -> 3521

R-w sense strand

Rank	Read number	Nucleotide sequence (5′ to 3′)	Position
1	1277	UUUUGGAUGAAAUGAGAAAGGC	3606 -> 3627
2	463	CUUUGUUCGAGCUCGUUUCAGC	3276 -> 3297
3	202	UUGGAACUCUCUAUUCUGGAUU	3454 -> 3475
4	192	AAGAAGGUUUAGGAACUACUGG	3636 -> 3657
5	184	GGCAUUUCUGAUUGGAACUCUC	3443 -> 3464
6	159	UUUGGAUGAAAUGAGAAAGGCC	3607 -> 3628
7	134	AAAUAAAGUUGGGCCUAAAGCC	3513 -> 3534
8	110	UUGGACCAAGUUGAAAUAAAGU	3500 -> 3521
9	85	CAAAGAUGUUCCUGGGCUGAUC	3376 -> 3397
10	83	UGUUCGAGCUCGUUUCAGCAGC	3279 -> 3300

b

J-w antisense strand

Rank	Read number	Nucleotide sequence (5′ to 3′)	Position
1	13486	UGAAACGAGCUCGAACAAAGGC	3274 -> 3295
2	9274	UCUGAACAAACAACAAGGACUC	3126 -> 3147
3	4835	GGAGAAGAGUUUGGGCUGCUGA	3293 -> 3314
4	1964	UUUGAGUAAGUGGAAUGUAAGC	3358 -> 3379
5	1563	UUGGCUAACCGAAGAACCGUGC	3075 -> 3096
6	1436	CUGAAACGAGCUCGAACAAAGG	3275 -> 3296
7	788	ACCGCGGUGAUUUCUGAACAAA	3138 -> 3159
8	594	UGCUGAAACGAGCUCGAACAAA	3277 -> 3298
9	422	UGACCGCGGUGAUUUCUGAACA	3140 -> 3161
10	419	UUAAGUUUCUCGGGCUUUAGGC	3525 -> 3546

R-w antisense strand

Rank	Read number	Nucleotide sequence (5′ to 3′)	Position
1	8199	UGAAACGAGCUCGAACAAAGGC	3274 -> 3295
2	2273	UCUGAACAAACAACAAGGACUC	3126 -> 3147
3	1051	UUUGAGUAAGUGGAAUGUAAGC	3358 -> 3379
4	805	UAGCAACACUGUGGAGGACAAC	3716 -> 3737
5	647	CUGAAACGAGCUCGAACAAAGG	3275 -> 3296
6	431	UUGGCUAACCGAAGAACCGUGC	3075 -> 3096
7	412	GGAGAAGAGUUUGGGCUGCUGA	3293 -> 3314
8	369	UGCUGAAACGAGCUCGAACAAA	3277 -> 3298
9	188	ACCGCGGUGAUUUCUGAACAAA	3138 -> 3159
10	182	UUAAGUUUCUCGGGCUUUAGGC	3525 -> 3546
