# Supplementary figures and images for: Deep sequencing uncovers commonality in small RNA profiles between transgene-induced and naturally occurring RNA silencing of chalcone synthase-A gene in petunia
Source: BMC Genomics. 2013 Jan 30;14:63. doi: 10.1186/1471-2164-14-63 (PMC3608071; doi:10.1186/1471-2164-14-63)

Figure S2

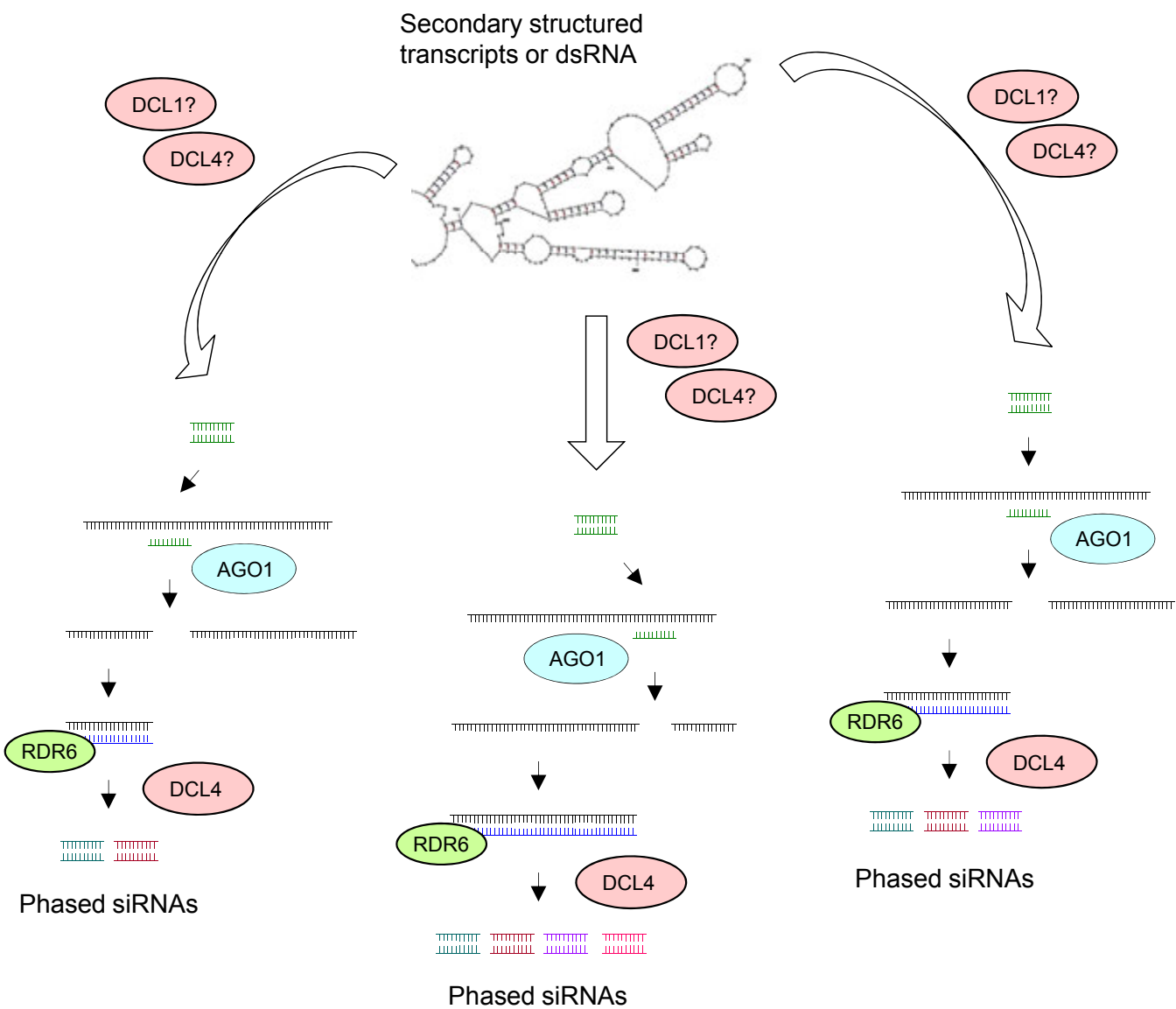

Supplement: Additional file 2 Figure S2 — RNA cleavage at various sites that initiate production of siRNA can be a feature of siRNA production common to cosuppression and naturally occurring RNA silencing of the CHS-A gene. siRNAs are produced from DCL cleavage of secondary-structured nascent CHS-A transcripts or dsRNAs produced by RDR6 orthologue(s) from the nascent transcripts. These siRNAs then cleave the CHS-A RNA at the target site with AGO, which triggers RDR6-mediated dsRNA production and subsequent DCL cleavage that produces phased siRNAs. [file 1471-2164-14-63-S2.pdf]

Figure S3

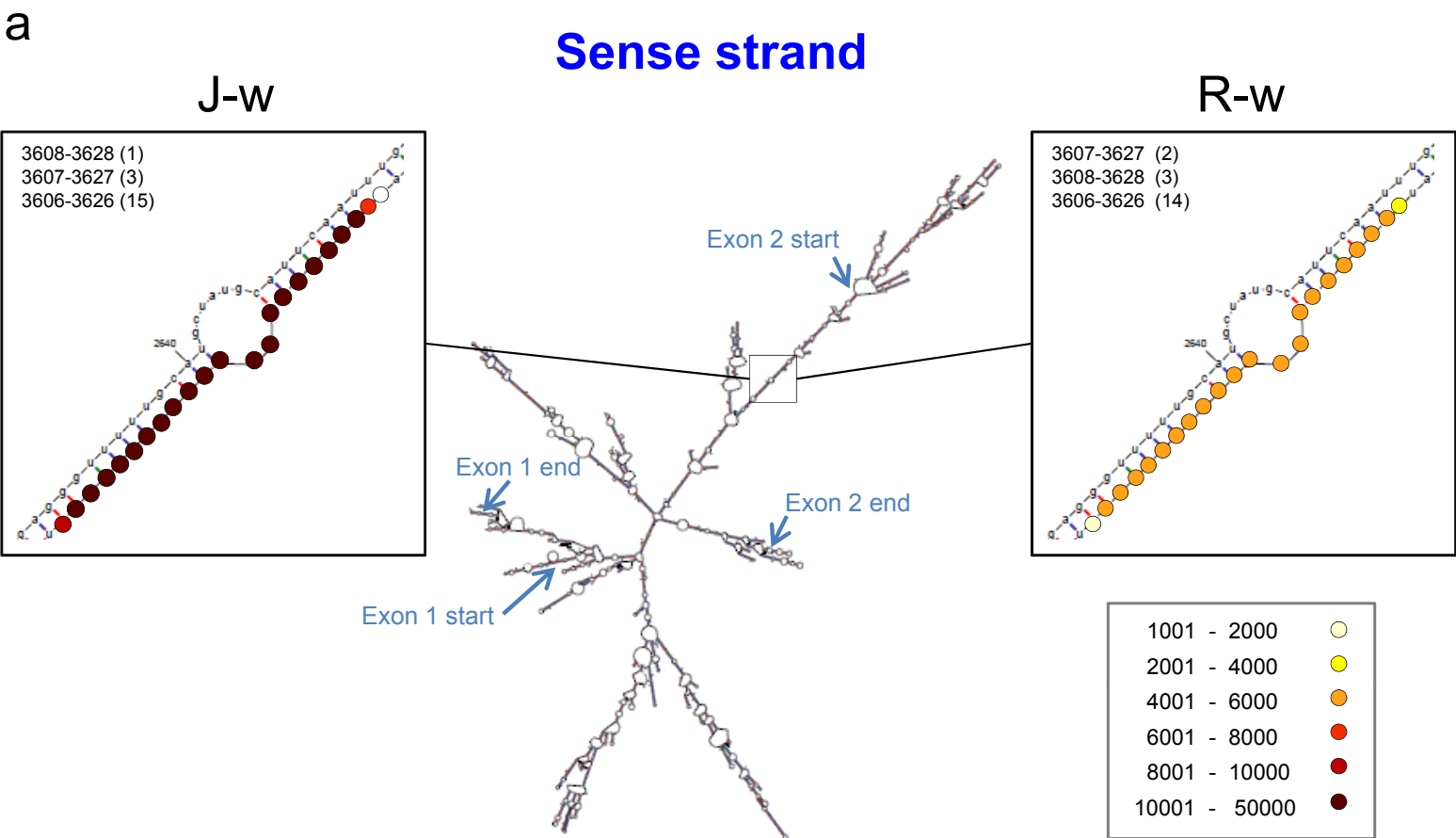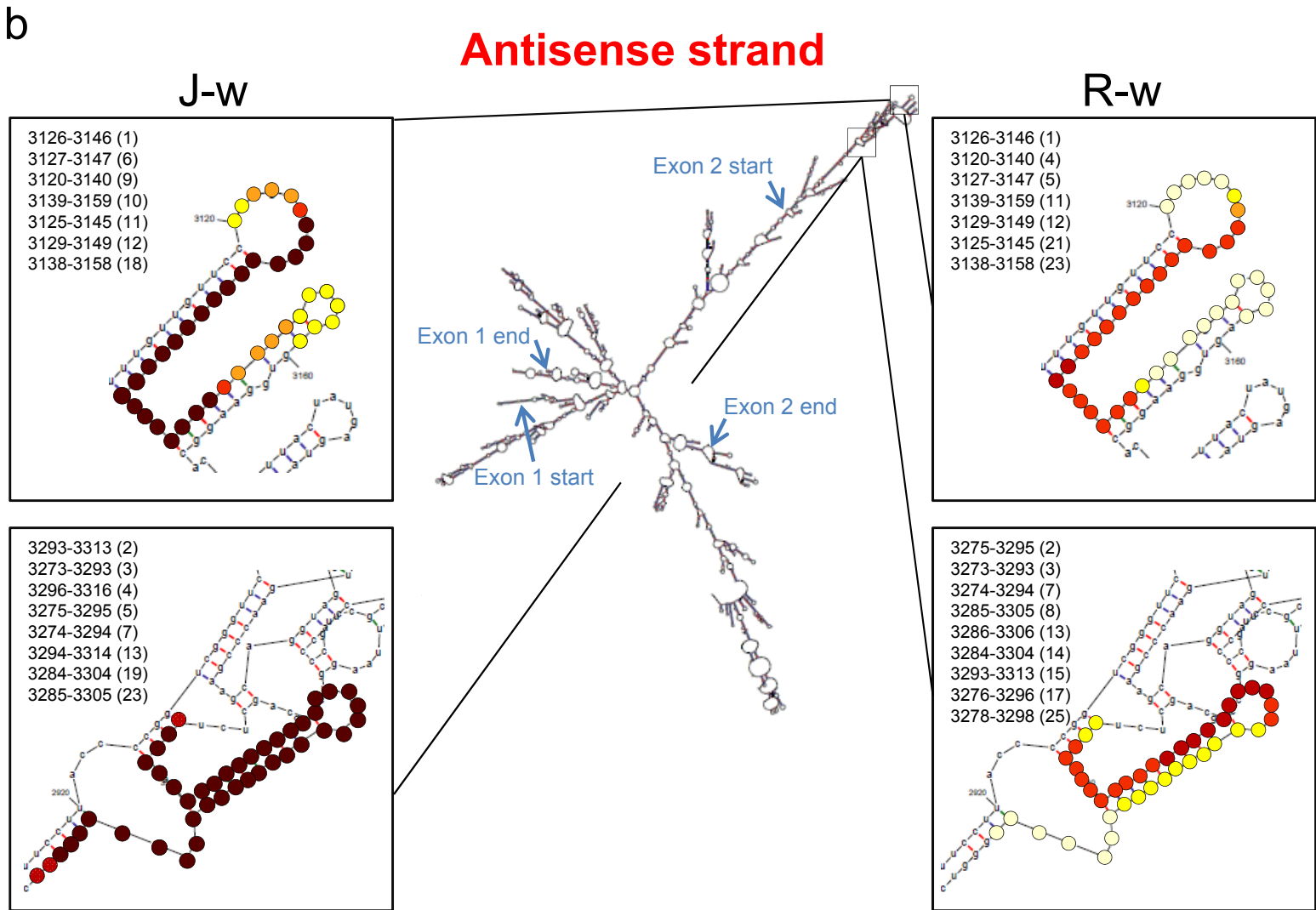

Supplement: Additional file 3 Figure S3 — Commonality of the siRNA hot spots between J-type and Red Star petals. Abundant siRNAs in the white tissues of J-type and Red Star petals are mapped on a secondary structure (a) and antisense (b) strands predicted by m-fold. Close ups of major hot spots are shown in windows, in which positions of nucleotides corresponding to siRNAs are marked by circles. Darker colors represent more total reads of siRNAs that contain the nucleotide. Mapped positions of siRNAs often overlapped, so that neighboring nucleotides had different colors. Nucleotide positions of abundant siRNAs mapped in each region are listed in the corresponding windows. Ranks of siRNA according to read number (see Figure 7) are in parentheses. [file 1471-2164-14-63-S3.pdf]
